# Corona-Pandemie und die Stabilität des Bankensystems: Ursachen in der Realwirtschaft kurieren

**DOI:** 10.1007/s10273-020-2783-3

**Published:** 2020-11-20

**Authors:** Markus Demary, Michael Hüther

**Affiliations:** 1 Institut der deutschen Wirtschaft Köln e.V., Konrad-Adenauer-Ufer 22, 50668 Köln, Deutschland; 2 Institut der deutschen Wirtschaft Köln e.V., Konrad-Adenauer-Ufer 21, 50668 Köln, Deutschland

**Keywords:** E32, G21, G28

## Abstract

Dass die Corona-Pandemie die Bilanzqualität von Banken und Finanzintermediären verschlechtern könnte, wurde zwar schon befürchtet, jedoch stand die Bankenstabilisierung bisher nicht im Mittelpunkt der wirtschaftspolitischen Maßnahmen, welche die Auswirkungen der Corona-Pandemie überwinden sollen. Vielmehr hatte sich der Lockdown auf mehrere Branchen der Realwirtschaft ausgewirkt. Besonders betroffen waren bisher der stationäre Einzelhandel, die Tourismusbranche, das Gastgewerbe, aber auch die Industrie. Die Automobilbranche hatte sogar vorübergehend die Produktion vollständig stillgelegt. Inwieweit könnte die Corona-Pandemie nun den Bankensektor destabilisieren?

## Abstract (en)

Although it was feared that the corona pandemic could worsen the balance sheet quality of banks and financial intermediaries, bank stabilisation has not yet been the focus of economic policy measures to overcome the effects of the corona pandemic. Rather, the effects of the lockdown have impacted several sectors of the real economy. So far, stationary retail trade, the tourism sector and hotels and restaurants have been hit by the lockdown. In addition, industry has been particularly affected by an interruption in supply chains. The automotive industry even temporarily halted production completely. The question now is to what extent the corona pandemic may destabilise the banking sector.

Nachdem sich die Lieferketten weitestgehend wiederhergestellt haben, trifft der Corona-Schock die Wirtschaft über eine plötzliche Zunahme der Ersparnis der Haushalte durch deren Konsumzurückhaltung bei Gütern und Dienstleistungen, die mit sozialem Kontakt verbunden sind. Infolgedessen erleiden Unternehmen Umsatzverluste und müssen zur Deckung ihrer laufenden Kosten kurzfristige Kredite aufnehmen, wodurch sich ihre Bilanzqualität verschlechtert. Zwar ergeben sich aus den Corona-Hilfskrediten, da diese durch den Staat besichert sind, keine direkten Ausfallrisiken für die Banken. Doch sind durch die gestiegene Verschuldung Ausfälle bei den Altkrediten zu befürchten. So weist die Bundesbank in ihrem Finanzstabilitätsbericht von 2019 darauf hin, dass der lange Aufschwung dazu geführt hat, Kredite unter lockeren Konditionen zu vergeben und in diesen Zeiten weniger Risikovorsorge zu betreiben (Deutsche Bundesbank, 2019a). Für die Kalibrierung der Risikomodelle werden in der Regel Daten aus den letzten zehn Jahren verwendet. Eine Krise dieses Ausmaßes konnte nicht in die Kalibrierung der Risikomodelle eingehen, wodurch Kreditrisiken unterschätzt wurden. Geraten die Unternehmen nun durch den Corona-Schock in Schieflage, kann es zu Kreditausfällen in den Altbeständen, besonders bei den längerfristig vergebenen Krediten kommen. Das ist umso wahrscheinlicher, je mehr die laxeren Kreditbedingungen ohnehin schon fragwürdig gewordene Geschäftsmodelle stabilisierten, die unter Corona-Bedingungen aber kollabieren mussten.

**Dr. Markus Demary** ist Senior Economist für Geldpolitik und Finanzmarktökonomik am Institut der deutschen Wirtschaft (IW).

**Prof. Dr. Michael Hüther** ist Direktor des Instituts der deutschen Wirtschaft (IW) in Köln und Hochschullehrer an der European Business School in Oestrich-Winkel.

Eine zusätzliche Bankenkrise würde den Aufschwung nach Corona deutlich bremsen. Schularick et al. (2020) schlagen aus diesem Grund eine vorsorgliche Rekapitalisierung der Großbanken in der Eurozone über den European Stability Mechanism (ESM) vor. Wie hoch das Risiko einer Bankenkrise hierzulande ist, wird nachfolgend für alle drei Säulen des deutschen Bankensystems analysiert. Denn es ist zu beachten, dass die Banken aufgrund ihrer unterschiedlichen Ausrichtung auch von unterschiedlichen Risiken der Corona-Krise betroffen sind: Unternehmensinsolvenzen in besonders von Corona betroffenen Branchen, Kreditausfälle in der Schiff- und Flugzeugfinanzierung sowie Verwerfungen auf den Märkten für Staatsanleihen.

Tabelle 1
**Strukturkennzahlen deutscher Banken**

| | Großbanken | Weitere Privat-banken | Landesbanken | Sparkassen | Kreditgenossen-schaften |
|---|---|---|---|---|---|
| Zahl | 4 | 146 | 6 | 378 | 841 |
| Kreditvolumen pro Bank in Mrd. Euro | 138,2 | 3,6 | 61,5 | 2,4 | 0,8 |
| Anleihevolumen pro Bank in Mrd. Euro | 32,2 | 1,0 | 17,8 | 0,5 | 0,2 |
| Eigenkapitalquote in % | 3,7 | 7,6 | 4,9 | 8,9 | 8,4 |
| Eigenkapitalrentabilität nach Steuern in % | 1,2 | 1,9 | -3,9 | 4,8 | 5,5 |

Quelle: Deutsche Bundesbank (2019b); eigene Berechnungen.

## Die Transmission des Corona-Schocks auf die Banken

Für die Transmission des Corona-Schocks von der Realwirtschaft auf die Banken sind grundsätzlich drei Mechanismen möglich (Demary und Hüther, 2020):

- *Unternehmensinsolvenzen*: Viele Unternehmen haben während des Corona-Lockdowns Umsatzausfälle erlitten. Dies betrifft vor allem Unternehmen aus den Branchen, in denen soziale Kontakte eine große Rolle spielen, wie das Gastgewerbe, die Tourismusbranche oder der Messebau. Aber auch die Unternehmen des Verarbeitenden Gewerbes mussten durch den Zusammenbruch von Lieferketten Umsatzausfälle hinnehmen. Um die laufenden Kosten kurzfristig decken zu können, mussten sich viele Unternehmen kurzfristig stärker verschulden oder Eigenkapital verzehren. Da das Fremdkapital nicht investiert wird, sondern lediglich der Deckung der laufenden Kosten dient, sinkt die Bonität der Unternehmen, und damit steigt das Kreditrisiko für die Banken. Haben diese nun Kredite an Unternehmen mit zu geringen Liquiditäts- und Eigenkapitalpuffern verliehen, so drohen Kredite notleidend zu werden. Dies gilt umso mehr, wenn zudem Eigenkapital eingesetzt wurde.

- *Schiffs- und Flugzeugfinanzierung*: Vom Einbruch des Tourismus unmittelbar betroffen sind die Kreuzfahrtbranchen und die Fluggesellschaften. In beiden Branchen müssen Investitionen mit hohen Volumina an Fremdkapital finanziert werden. Dabei dienen die Schiffe und die Flugzeuge zwar als Kreditsicherheiten. Jedoch können diese Kreditsicherheiten bei Kreditausfall nur bedingt veräußert werden, wenn sich die gesamte Branche in der Krise befindet. Zudem besteht die Gefahr, dass die Banken die Kreditausfallwahrscheinlichkeit als zu gering angenommen und deshalb zu wenig Eigenkapital gegen Verluste aus diesen Krediten aufgewendet haben, da die Kreditausfälle in diesem Segment in den vergangenen zehn Jahren sehr gering waren.

- *Verwerfungen auf den Märkten für Staatsanleihen*: Um ihre Wirtschaft vor einem Zusammenbruch zu bewahren, mussten sich die Staaten verschulden. Aufgrund des Niedrigzinsniveaus und einer hohen Nachfrage der Finanzinvestoren nach liquiden und risikoarmen Staatsanleihen konnten viele Staaten sich trotz Krise noch recht gut finanzieren. Dies wurde auch durch expansive Geldpolitik ermöglicht. Eine anhaltende Wirtschaftsschwäche kann aber dazu führen, dass die Staatseinnahmen sinken, was die Finanzmarktteilnehmenden die Rückzahlung ihrer Anleihen anzweifeln lässt. Ein Downgrade eines oder mehrerer größerer Staaten würde dann zu Verlusten bei den Banken führen, die diese Staatsanleihen halten. Da Banken für europäische Staatsanleihen kein Eigenkapital vorhalten müssen, könnte eine Staatsschuldenkrise zu einer Schieflage bei Banken führen.

## Risikofaktoren für unterschiedliche Bankengruppen

Es wirken sich nicht alle drei Risikofaktoren gleichermaßen auf die Banken in Deutschland aus, da hier das Bankensystem aus unterschiedlichen Bankengruppen besteht, innerhalb derer sich die Institute durch ihre Größe und ihre geschäftliche Ausrichtung unterscheiden. Die vier Großbanken verfügen im Durchschnitt über ein Kreditvolumen von 138,2 Mrd. Euro pro Bank, gefolgt von den sechs Landesbanken mit einem durchschnittlichen Kreditvolumen von 61,5 Mrd. Euro pro Bank. Deutlich kleiner sind hingegen die 378 Sparkassen und 841 Kreditgenossenschaften mit einem durchschnittlichen Kreditvolumen von 2,4 Mrd. und 0,8 Mrd. Euro pro Bank (vgl. Tabelle 1).

Der Vergleich der Kreditvolumina deutet darauf hin, dass die großen Industriefinanzierungen über die Großbanken und die Landesbanken getätigt werden, während die Sparkassen und die Kreditgenossenschaften regionale kleine und mittlere Unternehmen finanzieren. Die unterschiedliche Ausrichtung zeigt sich auch bei den Anleihevolumina. Großbanken halten im Durchschnitt Anleihen im Wert von

32,2 Mrd. Euro, gefolgt von den Landesbanken mit 17,8 Mrd. Euro pro Bank. Im Vergleich dazu halten die Sparkassen und die Kreditgenossenschaften 0,5 Mrd. und 0,2 Mrd. Euro an Anleihen pro Institut. Während die Großbanken und die Landesbanken vor allem Staatsanleihen und Unternehmensanleihen halten, haben die Sparkassen und Kreditgenossenschaften eher Kommunalanleihen in ihren Bilanzen. Daraus lassen sich die Risikofaktoren ableiten. Während sich die Großbanken und die Landesbanken den Risiken der globalen Kapitalmärkte ausgesetzt haben, sind die Sparkassen und die Kreditgenossenschaften vor allem durch regionale Risiken betroffen. Somit wären die Großbanken und die Landesbanken infolge des Corona-Schocks vor allem durch eine Staatsschuldenkrise und Ausfälle in der Schiffsfinanzierung sowie der Flugzeugfinanzierung oder von Insolvenzen größerer Unternehmen betroffen, während die Insolvenzen von kleinen und mittleren Unternehmen die Sparkassen und die Kreditgenossenschaften betreffen würden.

## Profitabilität und Verlustabsorptionsfähigkeit

Nicht nur bei der Kreditvergabe zeigen sich Unterschiede zwischen den Bankengruppen, sondern auch bei der Profitabilität und der Verlustabsorptionsfähigkeit (vgl. Tabelle 1). Sparkassen und Kreditgenossenschaften sind deutlich profitabler und stärker kapitalisiert als Großbanken und Landesbanken. Während die Sparkassen und die Kreditgenossenschaften eine Eigenkapitalrentabilität nach Steuern von 4,8 % und 5,5 % aufweisen, liegt die Eigenkapitalrentabilität bei den Großbanken bei 1,2 % und bei den Landesbanken bei -3,9 %. Vorteilhaft ist die höhere Profitabilität, denn sie ermöglicht es den kleineren Banken, über die Einbehaltung ihrer Gewinne Eigenkapital aufzubauen. So betragen die bilanziellen Eigenkapitalquoten 8,9 % bei den Sparkassen und 8,4 % bei den Kreditgenossenschaften, während die Eigenkapitalquote der Großbanken bei nur 3,7 % und die der Landesbanken bei nur 4,9 % liegt. Vor der globalen Finanzkrise wiesen die Sparkassen und Kreditgenossenschaften noch geringere bilanzielle Eigenkapitalquoten auf als die Großbanken (Demary und Hüther, 2020).

Damit scheinen die regional ausgerichteten Sparkassen und Kreditgenossenschaften in Bezug auf die Profitabilität und die Verlustabsorptionsfähigkeit gut gegen steigende Kreditausfälle durch die Corona-Pandemie gewappnet zu sein (Demary und Hüther, 2020). Dies bedeutet allerdings nicht, dass es nicht doch innerhalb der Bankengruppen zu einer Schieflage bei einzelnen Instituten kommen kann. Jedoch verfügen beide Bankengruppen über eigene interne Aufsichts- und Sanierungsmechanismen (Institutssicherung), mit denen sich Schieflagen einzelner Institute rechtzeitig entdecken lassen. Zudem bestehen Instrumente, um

Tabelle 2
**Kreditvergabe der Banken nach Branchen**
Anteile in %

| | Großbanken | Regionalbanken | Landesbanken | Sparkassen | Kreditgenossenschaften |
|---|---|---|---|---|---|
| Weniger stark von der Corona-Pandemie betroffene Branchen | | | | | |
| Industrie | 18,9 | 8,0 | 11,0 | 8,3 | 7,0 |
| Grundstückswesen/ Wohnungswesen | 22,4 | 21,5 | 25,9 | 33,9 | 29,2 |
| Datenverarbeitung | 12,3 | 8,1 | 7,8 | 8,8 | 9,2 |
| Bauwirtschaft | 2,4 | 3,6 | 1,5 | 7,0 | 7,1 |
| Energie | 5,4 | 11,6 | 13,7 | 5,9 | 5,2 |
| Finanzwirtschaft | 11,7 | 10,1 | 9,4 | 12,1 | 16,5 |
| Weitere Dienstleistungen | 11,0 | 15,2 | 18,5 | 7,9 | 3,6 |
| Summe | 84,1 | 78,1 | 87,9 | 84,1 | 77,8 |
| Stärker von der Corona-Pandemie betroffene Branchen | | | | | |
| Landwirtschaft | 0,8 | 3,0 | 1,2 | 2,5 | 8,7 |
| Handel | 10,7 | 13,3 | 5,0 | 8,2 | 8,8 |
| Verkehr | 3,2 | 4,4 | 5,6 | 2,7 | 1,9 |
| Gastgewerbe | 1,2 | 1,2 | 0,3 | 2,5 | 2,8 |
| Summe | 15,9 | 21,9 | 12,1 | 15,9 | 22,2 |

Quelle: Demary und Hüther (2020) basierend auf Bundesbank (2019b).

Institute in Schieflage zu stabilisieren, beispielsweise durch eine Konsolidierung innerhalb der Bankengruppe.

Schwieriger ist die Lage allerdings bei den Groß- und Landesbanken, die in Bezug auf Profitabilität und Kapitalisierung vergleichsweise schwächer abschneiden. Zudem sind diese von Risikofaktoren betroffen, die durch größere Kreditvolumina und weniger Diversifikation gekennzeichnet sind, nämlich die Schiffs- und Flugzeugfinanzierung sowie die Staatsfinanzierung.

## Kreditexposures an besonders betroffene Branchen

Ein Blick auf die Kreditexposures der Bankengruppen nach Branchen zeigt (vgl. Abbildung 1), dass ein Großteil der Kredite an Unternehmen aus dem Bereich Grundstückswesen und Wohnen vergeben werden, deren Cash-flows von der Corona-Krise bisher weniger betroffen sind. Die Bundesbank weist aber in ihrem aktuellen Finanzstabilitätsbericht für 2020 darauf hin, dass es auch zu Kreditausfällen bei der Immobilienfinanzierung kommen könnte (Deutsche Bundesbank, 2020). Allerdings stehen hier werthaltige Kreditsicherheiten zur Verfügung, sodass sich die Ausfälle für die Banken in Grenzen halten werden. An das stark von Corona betroffene Gastgewerbe vergeben alle Bankengruppen vergleichsweise wenig Kredite. Größer sind die Exposures gegenüber den Un-

Abbildung 1
**Insolvenzen nach Wirtschaftsbereichen**
in % aller Insolvenzen, Januar 2008 bis März 2020

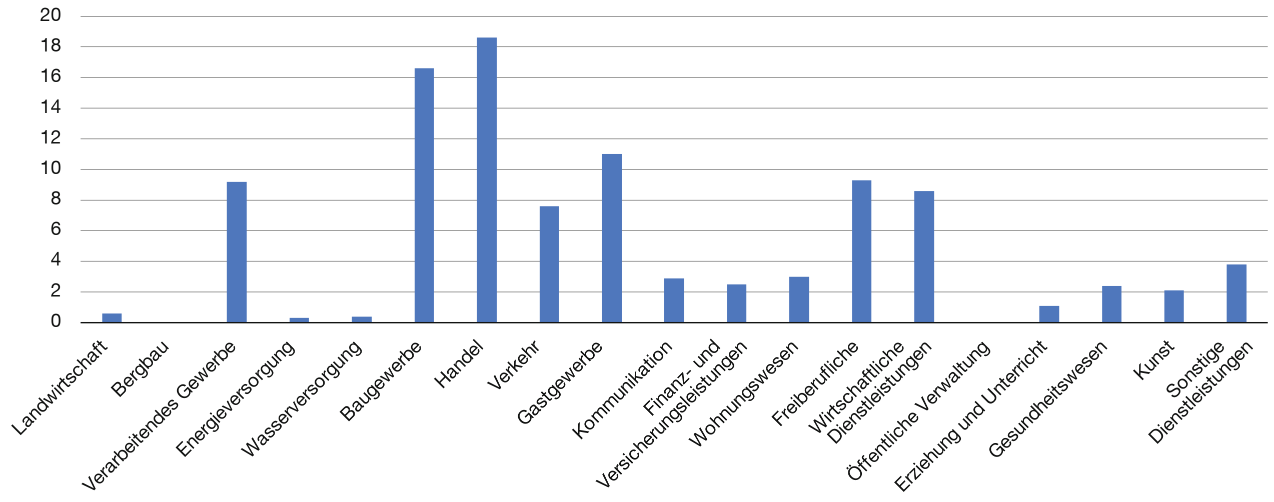

Quelle: Statistisches Bundesamt; eigene Berechnungen.

ternehmen aus dem Bereich Handel. Die Banken scheinen aber vergleichsweise gut diversifiziert zu sein (vgl. Tabelle 2). Aus der Corona-Betroffenheit der Branche kann aber nicht notwendigerweise geschlossen werden, dass diese Kredite auch alle ausfallen werden (Demary und Hüther, 2020).

## Insolvenzwahrscheinlichkeit der Unternehmen

Die Unternehmen der Realwirtschaft haben schon in den Jahren vor der globalen Finanzkrise ihre Liquiditätspuffer und ihre Eigenkapitalpuffer erhöht. Dabei stieg die bilanzielle Eigenkapitalquote des Unternehmenssektors von 20,4 % der Bilanzsumme (1997) auf 31,2 % (2017) an. Über denselben Zeitraum sank die Kreditnachfrage der Unternehmen, sodass der Anteil der Verschuldung bei Banken von 19,3 % der Bilanzsumme auf 10,5 % der Bilanzsumme fiel (Demary und Hüther, 2020). Überdurchschnittlich hohe Eigenkapitalquoten finden sich in den Industriebranchen, während die besonders durch Corona betroffenen Branchen, wie der Handel mit Kraftfahrzeugen, der Einzelhandel oder die Unternehmensdienstleistungen unterdurchschnittlich hohe Eigenkapitalquoten aufweisen und damit anfälliger für coronabedingte Schieflagen sind. Der Handel mit Kraftfahrzeugen und der Einzelhandel weisen zudem deutlich geringere liquide Mittel auf im Vergleich zu den Industrieunternehmen (Demary und Hüther, 2020).

Die höheren Eigenkapital- und Liquiditätspuffer spiegeln sich zum Teil auch in gesunkenen Insolvenzzahlen. Die geringen Insolvenzzahlen in Deutschland sind aber zusätzlich auf eine geringe Gründungsaktivität zurückzuführen, da junge Unternehmen eine viel höhere Insolvenzwahrscheinlichkeit haben als bereits auf dem Markt etablierte Unternehmen. Zudem gibt es in Deutschland mit dem Schutzschirmverfahren eine Alternative zum Insolvenzverfahren, sodass ein Unternehmen mit einem überzeugenden Sanierungsplan den Markt nicht notwendigerweise verlassen muss. Ein Vergleich der Insolvenzzahlen nach Branchen zeigt aber, dass die durch Corona besonders betroffenen Bereiche Handel und Gastgewerbe auch vor der Corona-Pandemie schon über hohe Insolvenzzahlen verfügt haben. Die höheren Kreditausfallraten in diesen Branchen sollten den Banken deshalb eigentlich bekannt und bei der Berechnung der Eigenkapitalunterlegung berücksichtigt worden sein.

## Risiken aus der Schiffs- und Flugzeugfinanzierung

Von der Pandemie ist besonders die Tourismusbranche betroffen und darüber die Schifffahrt sowie der Flugverkehr. Die Schiffsfinanzierung kam im Zuge der globalen Finanzkrise schon einmal unter Druck. Davon waren die Landesbanken besonders betroffen. Allerdings ist diese Krise schon mehr als zehn Jahre her. Möglicherweise sind die damaligen Ausfallraten in der Schiffsfinanzierung nach dem langen Aufschwung nicht mehr in den Risikoparametern der Banken enthalten. Die Flugzeugfinanzierung galt in der Vergangenheit als risikoarm, sodass hier womöglich die Kreditausfallwahrscheinlichkeiten unterschätzt wurden.

Das Eintreten einer Pandemie vom Ausmaß Covid-19 stellt ein sogenanntes Extremwertrisiko dar, auch Tail-Risiko genannt. Es handelt sich um ein Risiko mit einer sehr geringen Eintrittswahrscheinlichkeit, aber einer sehr hohen Verlustsumme bei Eintritt des Kreditausfalls. Seltene Ereignisse, wie eine globale Pandemie, sind oftmals in Datensätzen nicht enthalten und führen deshalb zur Unterschätzung der Eintrittswahrscheinlichkeit. Das Risiko, dass eine Pandemie den gesamten Flugverkehr lahmlegt und dass dies zu Kreditverlusten führen kann, wird bei Verwendung historischer Daten also unterschätzt. Neben der Eintrittswahrscheinlichkeit wurde womöglich auch die Höhe des Verlustes bei einem Kreditausfall unterschätzt. So kann in normalen Zeiten ein Flugzeug als Kreditsicherheit noch weiter veräußert werden. In einer Krise der gesamten Luftfahrt wird es allerdings schwierig, einen Käufer zu finden, was die Flugzeugfinanzierung derzeit riskanter werden lässt (Demary und Hüther, 2020). Im Vergleich zu Krediten im Bereich Handel oder Gastgewerbe fallen hier allerdings sehr große und nicht diversifizierte Kredite aus, wodurch das Eigenkapital der Banken empfindlich sinken kann. Da diese Art der großvolumigen Finanzierung vor allem von Groß- und Landesbanken in Form von syndizierten Krediten getätigt wird und eigentlich nicht von Sparkassen und Kreditgenossenschaften, fallen diese Risiken eher bei den großen und weniger bei den kleineren Banken an.

## Die Wahrscheinlichkeit einer Staatsschuldenkrise

Wie die Analyse der Strukturkennzahlen nach Bankengruppen gezeigt hat, wird eine Staatsschuldenkrise eher die Groß- und die Landesbanken treffen, während sie für die meisten Sparkassen und Kreditgenossenschaften keinen direkten Risikofaktor darstellen wird. Dies war auch schon während der Banken- und Staatsschuldenkrise von 2011 und 2012 der Fall. Die Corona-Pandemie könnte das Eintreten einer Staatsschuldenkrise in der Eurozone wahrscheinlicher machen, da sie sowohl auf den Zähler als auch auf den Nenner der Staatsschuldenquote wirkt (Demary und Hüther, 2020):

- Einkommensausfälle bei den privaten Haushalten und Umsatzausfälle bei den Unternehmen sowie den Selbstständigen führen zu geringeren Steuereinnahmen.

- Staatliche Liquiditätsmaßnahmen zur Vermeidung von Liquiditätsengpässen und Insolvenzen für Unternehmen und Selbstständige stellen zuerst einmal Staatsausgaben dar, auch wenn sie zu einem späteren Zeitpunkt zurückgezahlt werden müssen. Außerdem entstehen durch den Anstieg der Arbeitslosigkeit und die Subventionierung von Kurzarbeit Ausgaben für den Staat.

- Durch den rezessionsbedingten Einbruch des Bruttoinlandsprodukts (BIP) kommt es zu einem Anstieg der Staatsschuldenquote.

- Die Konjunkturprogramme wurden durch die Emission von Staatsanleihen finanziert. Der Effekt auf die Staatsschuldenquote hängt aber davon ab, wie sehr ein Einbruch des BIP durch das Konjunkturprogramm abgefedert werden kann.

- Aus einer steigenden Staatsschuldenquote leiten die Finanzmarktteilnehmenden häufig ab, dass die Rückzahlung der geliehenen Gelder riskanter wird. Als Reaktion darauf werden sie höhere Risikoprämien verlangen. Damit verteuern sich die nächsten Emissionen von Anleihen.

Da Staatsanleihen nicht nur ein Finanzierungsinstrument des Staates sind, sondern für den Ablauf von Finanztransaktionen eine herausragende Stellung haben, z. B. als Sicherheiten, aber auch für das Bilanzstrukturmanagement der Banken, hat ein plötzlicher Anstieg der Risikoprämien in der Regel einen starken Effekt auf den Finanzmarkt:

- Höhere Renditen auf Staatsanleihen führen zu höheren Renditen auf Unternehmensanleihen, was die Finanzierung der Unternehmen verteuert und deren Gewinn schmälert. Dadurch steigt ihr Kreditausfallrisiko. Für einige Unternehmen kann dies zu Zahlungsschwierigkeiten führen, was dann die Gewinne der Banken schmälert.

- Durch Rating-Downgrades stehen den Banken weniger werthaltige Anleihen als Sicherheiten bei Refinanzierungsgeschäften zur Verfügung. Für sie verteuert sich dadurch die Refinanzierung.

- Bei Wertverlusten von Staatsanleihen müssen Banken Abschreibungen auf ihr Eigenkapital vornehmen. Zur Wiederherstellung ihrer regulatorischen Eigenkapitalquoten verleihen sie dann nur noch sehr restriktiv an Unternehmen und Haushalte.

In der Regel halten Banken ein großes Volumen an Staatsanleihen ihres Heimatlandes (vgl. Abbildung 2); bei den italienischen Banken sind dies beispielsweise zwei Drittel des Anleihevolumens. Bei einem Rating-Downgrade italienischer Staatsanleihen werden die italienischen Banken deshalb recht stark belastet. Ähnlich sieht es bei den französischen und den spanischen Banken aus. Diese Länder sind besonders stark von der Corona-Pandemie betroffen, und ein zweiter Lockdown scheint hier möglich zu sein. Deutsche Banken halten 38 % ihrer Anleihen in Form von Bundesanleihen. Da diese recht wertsicher sind, wirkt sich

Abbildung 2

**Home Bias bei Staatsanleihen**

Volumen der Staatsanleihen in den Bankbilanzen, in Mrd. Euro, 4. Quartal 2019

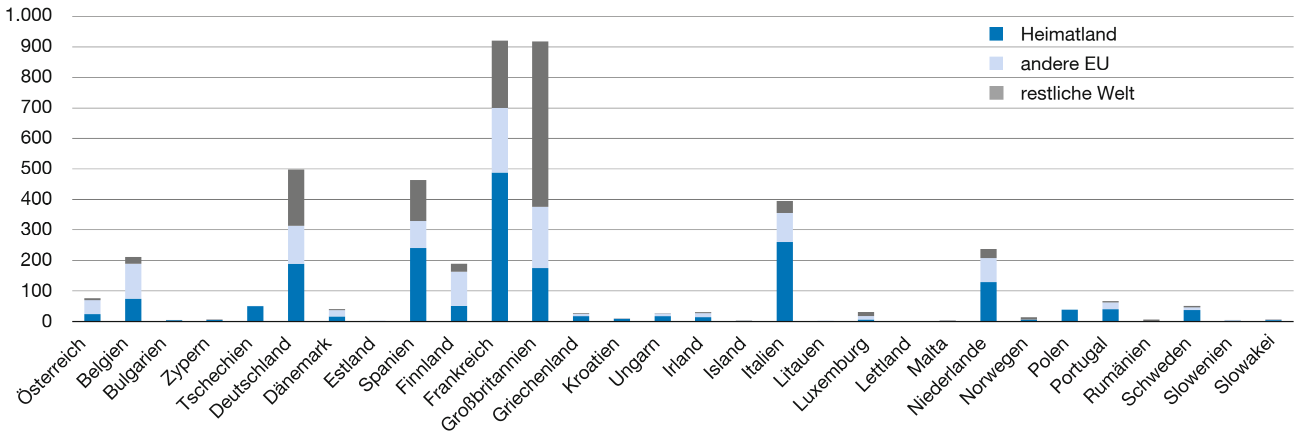

Quelle: European Banking Authority; eigene Berechnungen.

dieses hohe Volumen stabilisierend aus. Allerdings halten die deutschen Banken auch 25 % ihres Anleihevolumens in Form von europäischen Anleihen (124,5 Mrd. Euro). Bei einer Staatsschuldenkrise im Euroraum könnten sie deshalb durchaus höhere Verluste verzeichnen.

## Schlussfolgerung

Die Corona-Krise wird wahrscheinlich auch den Bankensektor erreichen. Die Frage ist nur, über welchen der drei denkbaren Transmissionskanäle. Die Großbanken und die Landesbanken wären vor allem durch einen Wertverlust bei Anleihen oder Großkrediten betroffen, sei es durch eine Staatsschuldenkrise im Euroraum oder durch Kreditausfälle im Schiffbau oder Flugzeugbau infolge eines Einbruchs in der Tourismusaktivität. Bei den Sparkassen und Kreditgenossenschaften werden sich eher viele kleinere Insolvenzen in Form von notleidenden Krediten wiederfinden. Die Analyse lässt aber vermuten, dass eine Staatsschuldenkrise und Kreditausfälle in der Schiffs- und Flugzeugfinanzierung für die Banken ein größeres Risiko darstellen als Insolvenzen bei Unternehmen aus anderen von Corona betroffenen Branchen. Das heißt, dass die Corona-Pandemie vor allem die weniger gut kapitalisierten Großbanken und Landesbanken treffen könnte und nur in geringerem Ausmaß Sparkassen und Kreditgenossenschaften.

Es scheint, als ob ein wirtschaftspolitischer Interventionsbedarf im deutschen Bankensektor aktuell trotz seiner strukturellen Schwächen erst einmal nicht besteht. Auch bei einem Anstieg der Insolvenzen um 35 %, wie von der Bundesbank erwartet, wäre das Finanzsystem noch nicht überfordert und im längerfristigen Vergleich noch weit von den Höchstständen zum Jahrtausendwechsel sowie zur Finanzkrise entfernt (Deutsche Bundesbank, 2020). Sollten Kredite tatsächlich notleidend werden, dann steht für deren Abbau zudem eine Guidance der EZB bereit, die in den vergangenen Jahren bereits erfolgreich angewendet wurde. Mit dem Single Resolution Mechanism der Bankenunion stehen Instrumente und finanzielle Mittel zur Verfügung, um Banken in Schieflage zu stabilisieren und abzuwickeln.

Die europäischen Risiken über den Markt für Staatsanleihen der Eurozonen-Staaten dürften durch die Beschlüsse des Europäischen Rates vom 20. Juli 2020 sowie die bereits im März von der EZB getroffenen geldpolitischen Entscheidungen angemessen adressiert sein. Eine prophylaktische Rekapitalisierung im großen Stil durch ESM-Mittel, wie von Schularick et al. (2020) vorgeschlagen, scheint noch nicht angemessen zu sein. Dafür spricht auch, dass sich die Kreditausfallswap-Prämien für europäische Unternehmen seit Mai 2020 wieder spürbar normalisiert haben, lediglich im High-Yield-Segment liegen diese noch spürbar über dem Vorkrisenniveau (Deutsche Bundesbank, 2020). Die ESM-Mittel sollten stattdessen zielgerichtet für eine mögliche Staatsschuldenkrise im Euroraum vorgehalten werden, um einer Bankenkrise durch Verwerfungen an den Märkten für Staatsanleihen entgegenzuwirken.

Passende wirtschaftspolitische Maßnahmen sollten deshalb erst einmal nicht auf die Banken abzielen, sondern auf die Verbesserung der Liquidität und der Bilanzen der Unternehmen der Realwirtschaft (Demary und Hüther, 2020):

- Die Tilgungsraten von Krediten könnten nach grundsätzlich positiver Einschätzung zur Tragfähigkeit des Geschäftsmodells steuerlich abzugsfähig gemacht werden. Die Stabilisierung der Realwirtschaft durch direkte und steuerliche Liquiditätshilfen, Kreditbürgschaften und Kreditprogramme sowie einmalige Zuschüsse stand zurecht seit Beginn des Pandemie-Schocks im Mittelpunkt der wirtschaftspolitischen Bemühungen.

- Mit dem Wirtschaftsstabilisierungsfonds sind auch gezielte Eigenkapitalhilfen für Unternehmen möglich, allerdings erst ab 250 Beschäftigten, 51 Mio. Euro Umsatz und 44 Mio. Euro Bilanzsumme. Es wäre zielführend, dieses unweigerlich verfahrensintensive Instrument durch eine erneute – und zwar systematische – Ausweitung des Verlustrücktrags zu einer Negativsteuer für Unternehmen zu erweitern.

- Überlegenswert erscheint auch, anstelle der Aussetzung der Insolvenzantragspflicht mehr Venture Capital für Neugründungen bereitzustellen, um dadurch den Strukturwandel zu befördern und krisenbedingte Verluste an Unternehmen kompensieren zu können. Da die Risikoaversion infolge dieser Pandemie eher steigen wird, ergibt sich hier ein möglicher Handlungsbedarf. Sofern der private Markt nicht schnell genug das notwendige Venture Capital hervorbringen kann, müsste eine Reform der Rahmenbedingungen geprüft werden, die hemmend auf Venture Capital Investments wirken. Sinnvoll ist ohne Zweifel der von der Bundesregierung ab Jahresanfang 2021 geplante „Stabilisierungs- und Restrukturierungsrahmen“, der Unternehmen in der Krise einen gesetzlich geschützten Rahmen bietet, in dem sie sich ohne ein Insolvenzverfahren nachhaltig restrukturieren können (Regierungsentwurf „Gesetz zur Fortentwicklung des Sanierungs- und Insolvenzrechts“ vom 14. Oktober 2020).

Auch wenn Corona für Branchen wie den Tourismus und das Gastgewerbe eine besondere Situation darstellt, kann und sollte der Staat nicht jede Insolvenz verhindern. Zunächst stellt eine Insolvenz nicht das Ende des Unternehmens dar, sondern es kann auch durch ein Sanierungsverfahren eine forcierte Neuaufstellung ermöglicht werden; zumal nach Inkrafttreten des Gesetzentwurfs. Zudem können nicht systemrelevante Unternehmen durch den Markt neu gegründet werden.

Ein Anstieg der Unternehmensinsolvenzen in von der Corona-Pandemie betroffenen Branchen sollte für die Banken erst einmal verkraftbar sein. Anders als in den Krisenjahren 2008 und 2009 gibt es aktuell keine Bankenkrise, und anders als zu Beginn der Bankenunion besteht aktuell wenig Unsicherheit über die Qualität der Bankbilanzen. Diese sind deutlich robuster als in der Zeit vor der globalen Finanzmarktkrise. Zudem sind die Bilanzen aktuell nicht durch toxische Wertpapiere, wie zu Zeiten der globalen Finanzkrise, vernetzt, d.h. die Banken halten aktuell keinen gemeinsamen Risikofaktor. Stattdessen sind die Kreditrisiken der Banken zurzeit gut diversifiziert.
